# Unusually Situated Binding Sites for Bacterial Transcription Factors Can Have Hidden Functionality

**DOI:** 10.1371/journal.pone.0157016

**Published:** 2016-06-03

**Authors:** James R. J. Haycocks, David C. Grainger

**Affiliations:** Institute of Microbiology and Infection, School of Biosciences, College of Life and Environmental Sciences, University of Birmingham, Edgbaston, Birmingham, United Kingdom; Hosei University, JAPAN

## Abstract

A commonly accepted paradigm of molecular biology is that transcription factors control gene expression by binding sites at the 5' end of a gene. However, there is growing evidence that transcription factor targets can occur within genes or between convergent genes. In this work, we have investigated one such target for the cyclic AMP receptor protein (CRP) of enterotoxigenic *Escherichia coli*. We show that CRP binds between two convergent genes. When bound, CRP regulates transcription of a small open reading frame, which we term *aatS*, embedded within one of the adjacent genes. Our work demonstrates that non-canonical sites of transcription factor binding can have hidden functionality.

## Introduction

A long standing view is that promoters, and transcription factor binding sites, should locate to the 5' end of an annotated gene [1]. Unexpectedly, recent genome-wide studies of RNA polymerase distribution, and transcription factor binding, have shown that the situation is more complex [2]. Notably, transcription factor binding sites are frequently found within genes or between convergent genes. For example, RutR (a regulator of pyrimidine catabolism) binds predominantly at sites within genes [3]. Similarly, LeuO (a regulator of leucine biosynthesis) binds to numerous targets between convergent genes [4]. A major challenge is to understand if, and how, such transcription factor binding sites contribute to gene regulation.

In this work we have focused on the the cAMP receptor protein (CRP). Widely conserved in bacteria, CRP is global regulator of transcription that responds to cAMP levels [1]. When bound to DNA, CRP regulates transcription by one of two distinct mechanisms. For example, at class I promoters, CRP binds upstream of the promoter -35 element and interacts with the C-terminal domain of the RNA polymerase α-subunit [5–7]. Conversely, at class II promoters, CRP binds close to the promoter -35 element and interacts with the N-terminal domain of the RNA polymerase α-subunit [8, 9]. In both cases, CRP stimulates transcription [5–9]. Recently, we have shown that CRP binds numerous sites that are not close to the 5' end of an annotated gene [10, 11]. In this work we have focused on one such atypical target; a CRP site between two convergent genes. Our data show that CRP activates expression of a small open reading frame (ORF) completely embedded within one of these convergent genes. Activation occurs by a standard class II CRP-dependent mechanism. Thus, although the genomic context of the CRP binding site is unusual, gene regulation proceeds via a well-defined mechanism.

## Methods and Materials

### Strains, plasmids and oligonucleotides

Strains and plasmids used are listed in Table 1. Oligonucleotides used are listed in Table 2.

**Table 1:** Strains and plasmids used in this study.

Strain or plasmid	Description	Source
**Strains**		
*Escherichia coli* M182	Δ*lac galK galU strA*	[39]
*Escherichia coli* M182*Δcrp*		[39]
**Plasmids**		
pRW50	Broad-host-range *lac* fusion vector for cloning promoters on *Eco*RI–*Hin*dIII fragments: contains the RK2 origin of replication and encodes TcR.	[40]
pSR	pBR322-derived plasmid containing an *Eco*RI–*Hin*dIII fragment upstream of the λ*oop* transcription terminator	[17]
pRW225	pRW50-derived plasmid in which the ribosome binding site upstream of *lacZ* has been deleted.	[23]
pDCRP	*crp* gene preceding its native promoter (located on *Eco*RI-*Sal*I flanked fragment). pBR322 derived. Encodes AmpR. ColE1 origin.	[41]

**Table 2:** Oligonucleotides used in this study.

Oligonucleotide name	Sequence (5'-3')	Source
Oligonucleotides used to generate P*aatS* derivatives		
*aatS*1 For	GGCTGCGAATTCATAAAGTGATAAAAATCACATAAAATTTTTATTAAAAGGATATAACCTTCATATCACTTGTAATTAAATTTGTGTCA	This study
*aatS*1 Rev	GCCCGAAGCTTCATGGATATACTTCTTAAGTATTATAAACAAGGTGGAACAGTTGTTATGGTAACCCATGACACAAATTTAATTACAA	This study
*aatS*2 Rev	GCCCGAAGCTTCATGGATATTGAAGAAAAGTATTATAAACAAGGTGGAACAGTTGTTATGGTAACCCATGACACAAATTTAATTACAA	This study
Oligonucleotides used for primer extension experiments		
D49724	GGTTGGACGCCCGGCATAGTTTTTCAGCAGGTCGTTG	[13]

### β-galactosidase assays

β-galactosidase assays were done according to the method of Miller [12]. Cells were grown in M9 minimal media supplemented with 1% fructose to stationary phase as specified in figure legends. Since P*aatS* was 2-fold more active in stationary phase cultures compared to log phase cultures the cells from the former phase of growth were used in all experiments. Values shown are the mean of three independent experiments. Error bars represent the standard deviation of three independent experiments. To calculate *P* we used a paired, two-tailed, student’s *t* test.

### Primer extension

Primer extension was done as described by Lloyd *et al*. [13]. Briefly, RNA was extracted from M182 cells (or the *Δcrp* derivative) harbouring pRW50 containing the *aatS*1 promoter fragment. Extractions were done using a Qiagen RNeasy mini kit according to the manufacturer’s instructions and residual DNA was removed using a Turbo DNA-free kit (Ambion). RNA integrity was then checked by visualisation following agarose gel electrophoresis. The ratio of absorbance at 260 nm and 280 nm was used to assess the purity of the RNA. Primer extension products were analysed on a 6% denaturing polyacrylamide gel alongside size standards generated by manual sequencing of M13 phage DNA. Full gel images are provided in the supporting information (S1 Fig).

### Proteins

CRP and σ^70^ proteins were purified as previously described [14, 15]. RNA polymerase core enzyme was purchased from Cambio, and was incubated at 37°C with a ten-fold molar excess of σ^70^ before use to generate RNA polymerase holoenzyme.

### DNAse I footprinting

DNA templates were generated by excision of *Aat*II-*Hin*dIII fragments from maxipreps of pSR plasmid containing the 134 bp *aatS*1 promoter fragment. The resulting 212 bp *Aat*II-*Hin*dIII fragment was labelled at the *Hin*dIII end using γ-dATP and T4 polynucleotide kinase. Footprinting reactions were done as previously described [15] in buffer containing 120 mM KCl, 100 μM EDTA, 20 mM Tris pH 7, 10 mM MgCl_2_ and ~10 nM of P*aatS* DNA. Reactions also containing 12.5 μg ml^-1^ Herring Sperm DNA as a competitive inhibitor. Footprints were analysed on denaturing 6% polyacrylamide gels alongside calibrating Maxam-Gilbert G/A ladders [16]. Full gel images are provided in the supporting information (S1 Fig).

### Multi-round *in vitro* transcription assays

*In vitro* transcription reactions were carried out as described by Savery *et al*., [14] using the method of Kolb *et al*. [17]. Supercoiled DNA was extracted from M182 cultures harbouring pSR containing the *aatS*1 fragment. Reaction buffer contained 20 mM Tris pH 7.9, 200 mM GTP/ATP/CTP, 10 mM UTP, 5 μCi (α^32^P) UTP, 500 mM DTT, 5 mM MgCl_2_, 100 μg ml^-1^ BSA and 0.2 mM cAMP. Template DNA (at a final concentration of 16 μg ml^-1^) was incubated with CRP at 37°C for 5 minutes in reaction buffer, prior to the addition of RNA polymerase holoenzyme to start the reaction. Full gel images are provided in the supporting information (S1 Fig).

### Bioinformatics

NCBI ORF finder was used to search for putative genes [18]. NCBI BLAST was used to search for *aatS* homologues. A list of DUF1602-containing proteins was obtained using the InterPro tool [19], the list is correct as of 14/01/16.

## Results

### Identification of promoter-like DNA sequence elements between two convergent genes

**Fig 1:**
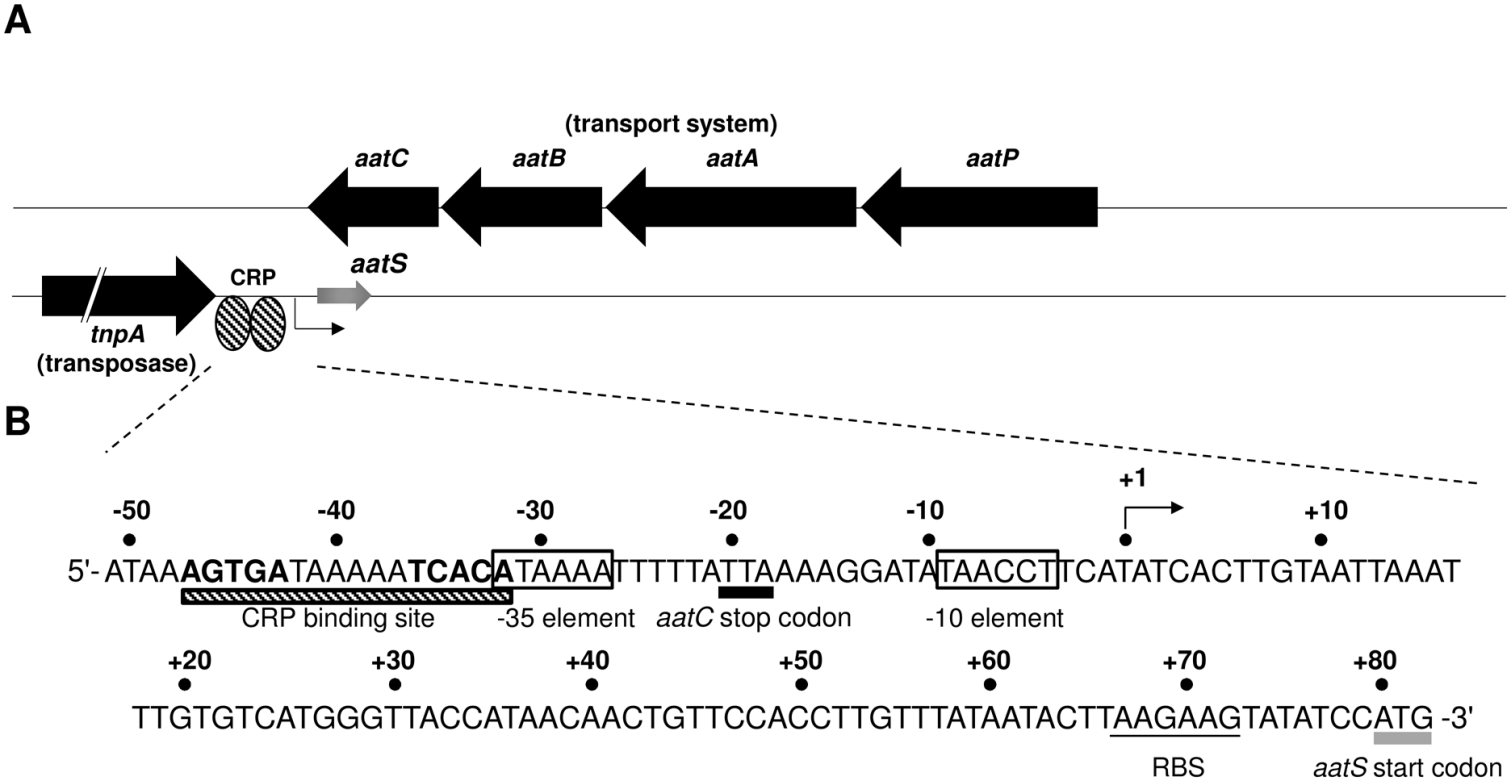
The *aatPABC* operon of ETEC H10407. Schematic of the *aatPABC* operon and adjacent *tnpA* gene. The two DNA strands are shown as black lines. Known genes are shown as black arrows and the predicted *aatS* gene as a grey arrow. Gene names are shown in italic and gene function in parenthesis. The position of a putative CRP binding site is indicated by striped ovals. **A.** Sequence if the *tnpA*-*aatC* intergenic region. The CRP site is highlighted as a striped rectangle with the two half sites highlighted bold. The start codon of the *aatS* open reading frame is highlighted with a grey rectangle. The transcription start site, as determined by mRNA primer extension is denoted “+1” and indicated by a bent arrow. Distances upstream (-) and downstream (+) of this start site are numbered. The -35 and -10 hexamers are boxed, and the ribosome binding site (RBS) is underlined.

The starting point for this work was our previous analysis of global CRP binding in enterotoxigenic *Escherichia coli* (ETEC) [11]. This work predicted a CRP target between the convergent genes *aatC* (encoding a subunit of a transport system) and *tnpA* (encoding a transposase). The genomic context of the CRP site is shown in Fig 1A and the surrounding DNA sequence is shown in Fig 1B. We searched the locus for promoters that may be under the control of CRP. A sequence (5'-TAACCT-3') that resembled a promoter -10 hexamer was found close to the CRP site within the *aatC* coding region (Fig 1B). A 189 bp ORF, which we named *aatS*, and a ribosome binding site (RBS) were also identified (Fig 1B). Note that *aatS* fully overlaps *aatC* but is in the opposite orientation. We considered that the overlap might result from *aatC* being annotated incorrectly. However, on close examination, this seems unlikely; there is no alternative stop codon within the *aatC* open reading frame. Furthermore, the full *aatC* sequence is conserved in numerous bacteria.

### P*aatS* is a CRP-dependent promoter *in vivo*

**Fig 2:**
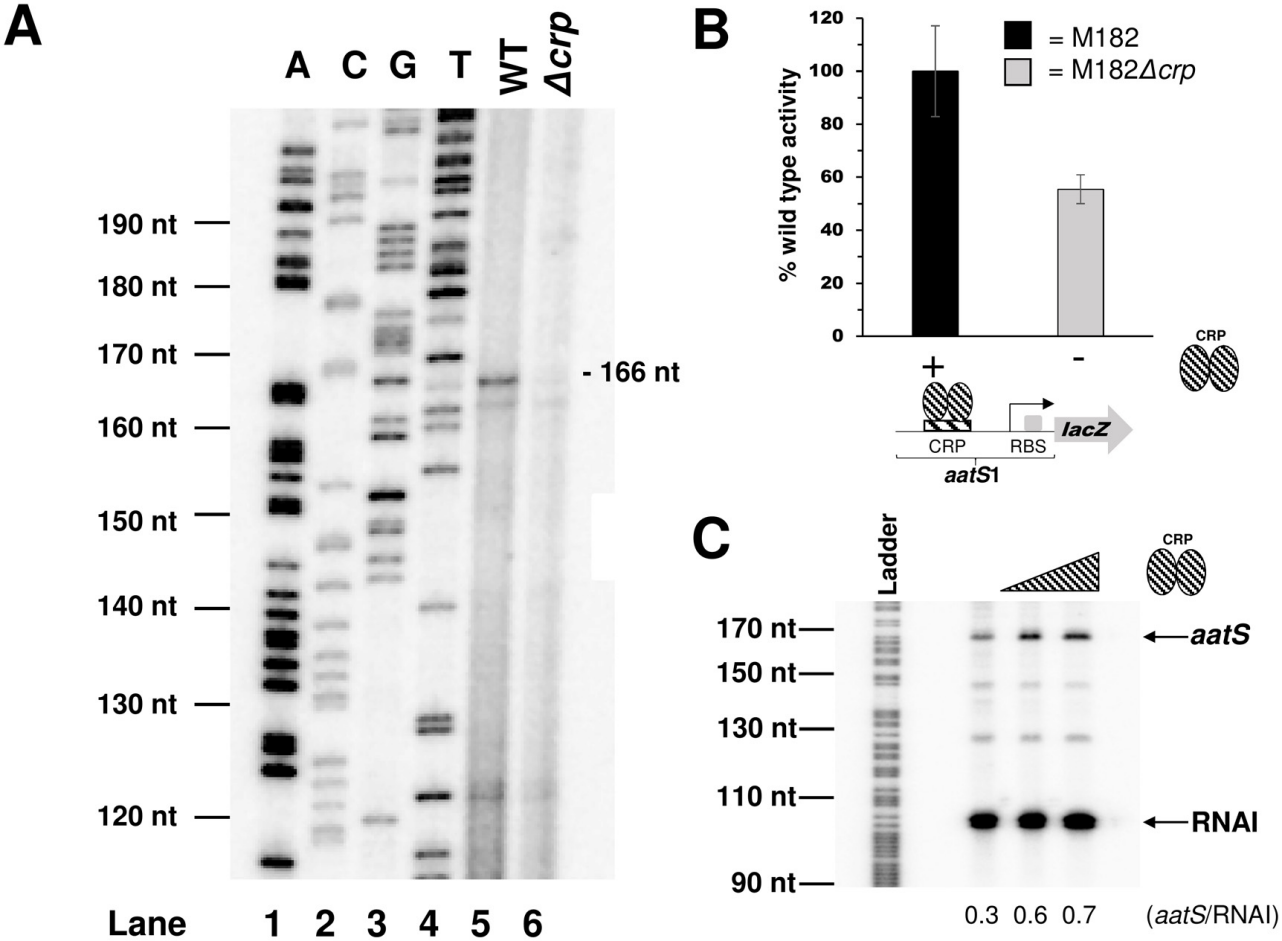
Characterisation of the P*aatS* promoter. **A.** Primer extension analysis of the *aatS* transcript. Lanes 1–4 on the gel are arbitrary size standards, used for calibration, generated by sequencing of M13mp18 phage DNA. Lane 5 shows the primer extension product generated using RNA from wildtype M182 cells carrying the *aatS*1::*lacZ* fusion. Lane 6 shows the primer extension product generated using RNA from M182*Δcrp* cells carrying the *aatS*1::*lacZ* fusion. The transcription start site is indicated in Fig 1B. **B.** β-galactosidase activity determined using lysates of M182 wildtype or M182Δ*crp* cells carrying P*aatS* cloned upstream of *lacZ* in plasmid pRW50. Values shown are percentages of activity observed in strain M182 (92 Miller units). We obtained 7 and 3 Miller units of activity from lysates of M182 or M182Δ*crp* carrying promoterless pRW50. Error bars represent the standard deviation of three independent experiments. **C.** Multi-round i*n vitro* transcription assay using P*aatS*. The *aatS*1 DNA fragment was cloned into pSR upstream of a *λoop* terminator. Purified, supercoiled pSR plasmid was incubated with purified CRP at 37°C, and the reaction started by the addition of 400 nM σ^70^- RNA polymerase holoenzyme. CRP concentrations are; 0 nM, 200 nM, or 400 nM. The 108 nt RNAI transcript from the pSR replication origin, and the 169 nt transcript from P*aatS*, are indicated. The gel is calibrated with an arbitrary G+A DNA sequencing reaction as a size standard.

Our next aim was to determine if the putative promoter upstream of *aatS* (P*aatS*) was functional. Thus, a 134 bp DNA fragment containing the sequence shown in Fig 1B, flanked by *Eco*RI and *Hin*dIII restriction sites, was generated. The DNA fragment, named *aatS*1, was cloned upstream of *lacZ* in plasmid pRW50 to create a P*aatS*::*lacZ* fusion. Next, *E*. *coli* M182 and the Δ*crp* derivative were transformed with the resulting plasmid. Transformants were cultured in liquid media and RNA was isolated as a template for primer extension. A 166 nucleotide primer extension product was observed using RNA from M182. Conversely, little extension product was observed using RNA from the Δ*crp* derivative (compare lanes 5 and 6 in Fig 2A). Thus, the *aatS* transcription start site (marked “+1” in Fig 1B) is located 4 bp downstream of the putative *aatS* promoter -10 hexamer. Furthermore, P*aatS* is poorly active in the absence of CRP. Consistent with our primer extension analysis, P*aatS* controlled expression of *lacZ* was also significantly reduced in cells lacking CRP (*P* = 0.018; Fig 2B).

### P*aatS* is a CRP dependent promoter *in vitro*

We next confirmed activation of P*aatS* by CRP using *in vitro* transcription assays. The *aatS*1 DNA fragment was cloned upstream of the *λoop* transcription terminator in plasmid pSR. Thus, the transcript generated from P*aatS* is expected to be 169 nucleotides (nt) in length. Note that, pSR also encodes RNAI; a 108 nt factor independent transcript derived from the plasmid replication origin. Thus, we observed two bands on denaturing PAGE gels (Fig 2C). As expected, synthesis of RNAI did not require CRP. However, production of the larger 169 nt *aatS* transcript was stimulated by CRP.

### CRP binds a site overlapping the P*aatS* -35 element

**Fig 3:**
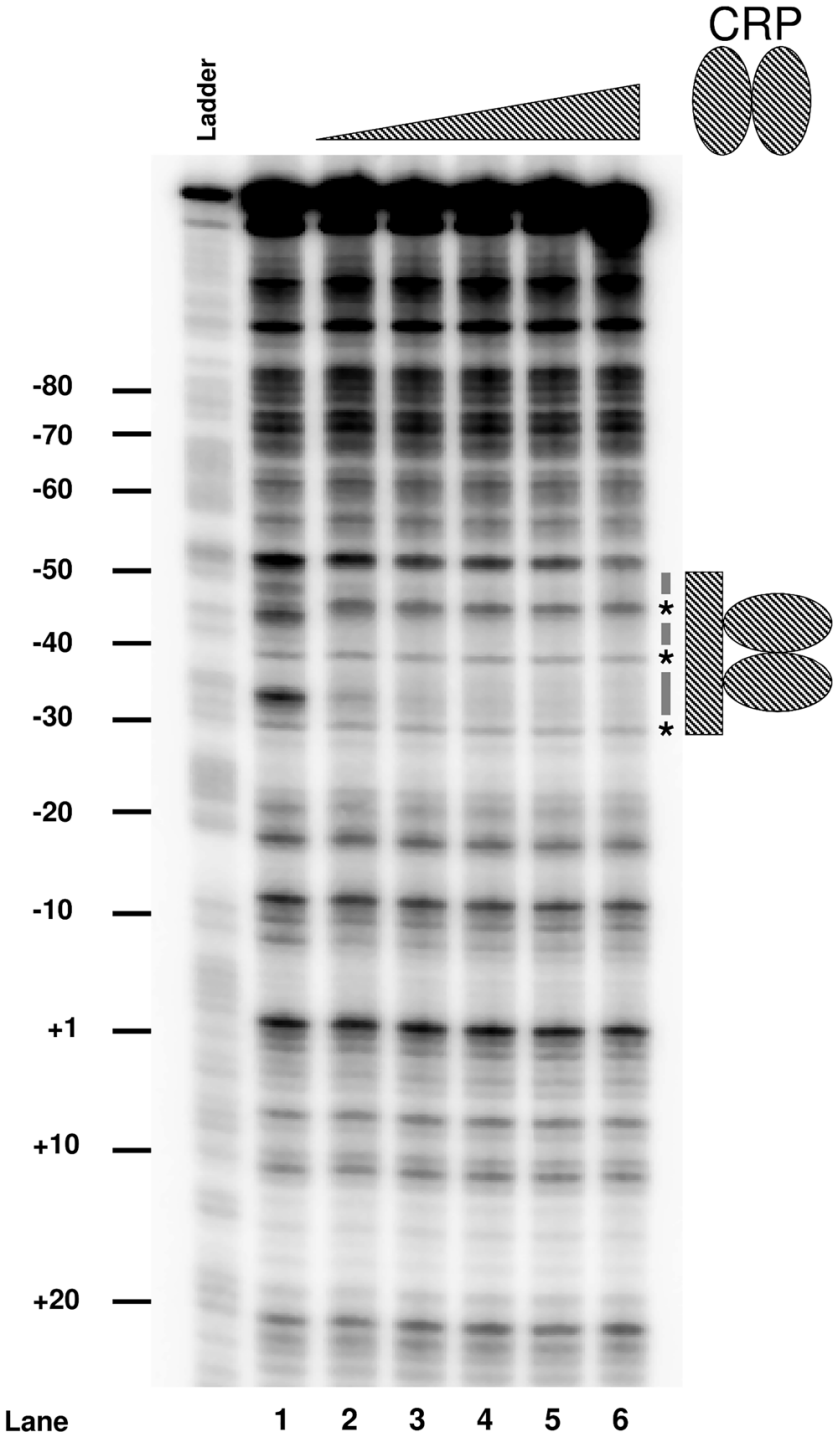
Binding of CRP to the P*aatS* region. DNAse I footprint analysis of the P*aatS* region. The lane labelled ‘G+A’ is a Maxim/Gilbert G+A sequencing reaction. Lane 1 shows the cleavage pattern obtained from *aatS*1 DNA digested with DNAse I in the absence of CRP. Lanes 2–6 show DNAse I cleavage patterns generated in the presence of increasing concentrations of CRP (0.35 μM, 0.7 μM, 1.05 μM, 1.4 μM and 2.1 μM). The predicted CRP site is indicated by a hashed grey bar. DNA protection is indicated by black dashes, hypersensitive bends are highlighted by stars.

To confirm CRP binding to the predicted target upstream of P*aatS* we used DNAse I footprinting. The result of the experiment is shown in Fig 3. Lane 1 shows the banding pattern resulting from DNAse I cleavage of *aatS*1 in the absence of CRP. Alterations to this pattern are evident in lanes 2–6 as increasing concentrations of CRP are added. Thus, CRP induced the appearance of three hypersensitive bands (starred) and protected the flanking DNA regions (underlined). The footprint aligns precisely with the predicted DNA target for CRP. The CRP site is 39.5 bases upstream of the P*aatS* transcription start site (Fig 1B). However, we note that the *aatS* mRNA start is 4 bp, rather than the usual 7 bp, downstream of the promoter -10 element. Hence, CRP is well positioned to interact with RNA polymerase via a standard class II mechanism.

### The *aatS* coding region is preceded by a functional ribosome binding site

**Fig 4:**
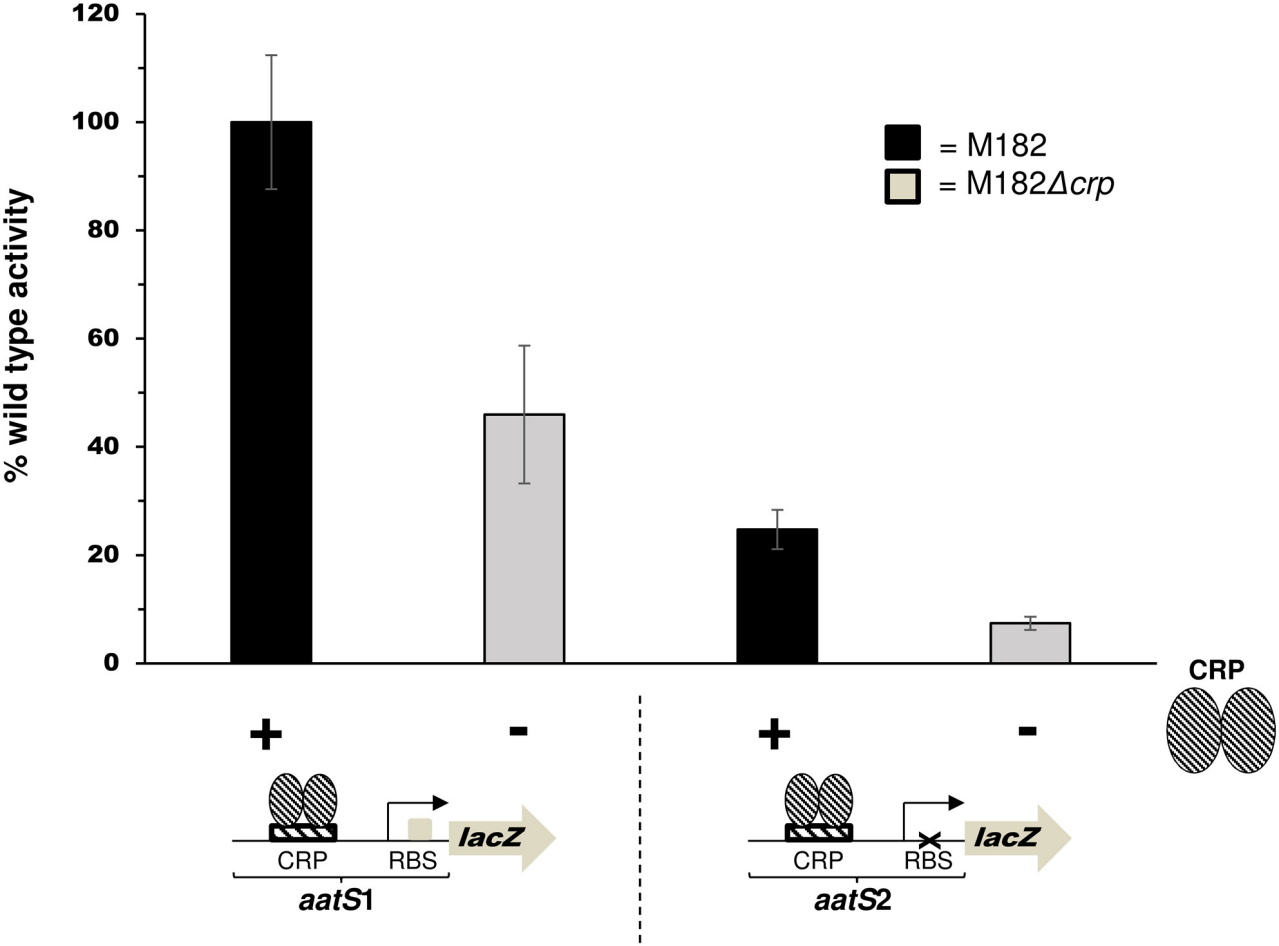
The *aatS* mRNA contains a functional ribosome binding site. The graph shows activity of different *aatS*:*lacZ* translational fusions. The wildtype ribosome binding site (5'-AAGAAG-3') in the *aatS*1 fragment was mutated to (5'-TTCTTC-3') in *aatS*2. LacZ activites was determined using the lysates of stationary phase M182 or M182*Δcrp*. In M182 cells *crp* was supplied in trans by plasmid pCRP that encodes *crp* under the control of its own promoter. Values shown are percentages of activity observed in strain M182 (5 Miller units). We obtained 0.25 and 0.26 Miller units of activity from lysates of M182 or M182Δ*crp*, carrying promoterless pRW225, respectively. Error bars represent the standard deviation of three independent experiments.

The predicted *aatS* ORF is located 82 bp downstream of the *aatS* transcription start site, within the coding region of *aatC*. We noticed that the 5' end of the *aatS* mRNA contains a sequence, 5'-UUAAGAAGU-3', that resembles a RBS (5'-UAAGGAGGU-3') [20, 21]. In addition, the predicted *aatS* RBS is located 6 bp upstream of the *aatS* start codon, a position suitable for translation initiation [22]. To determine if the RBS was functional, we created a translational *aatS*1::*lacZ* fusion and explored the effects of mutating the RBS on *lacZ* expression. Thus, we generated a derivative of the *aatS*1 fragment, called *aatS*2, where the sequence of the RBS was altered to 5'-UUUUCUUCA-3' (Fig 4). Both *aatS*1 and *aatS*2 were translationally fused to *lacZ* by cloning into pRW225 [23]. Resulting plasmids were used to transform M182 and the Δ*crp* derivative. LacZ activities were then determined in lysates of transformants grown in liquid culture (Fig 4). LacZ expression was significantly reduced when the *aatS* RBS was mutated (*P* = 0.011). Residual LacZ expression, driven by the *aatS*2::*lacZ* fusion in M182Δ*crp* cells, was not significantly different from background LacZ activity (*P* = 0.096). Background LacZ activity was obtained using M182Δ*crp* transformed with pRW225 having no promoter insert.

### AatS contains a conserved domain of unknown function

We cannot exclude the possibility that P*aatS* drives transcription of a small or antisense RNA. However, the presence of a functional RBS, appropriately positioned upstream of the *aatS* open reading frame, is consistent with the transcript being an mRNA. If this is the case, translation would result in the production of a 62 amino acid AatS peptide. To better understand AatS we used BLAST to identify 38 microbial proteins with a significantly similar sequence (E-value <10) in the NCBI blastp database. The search also reveals that AatS shares 67% identity with a conserved domain of unknown function (DUF1602, E-value: 4.07e^-7^). Genes encoding DUF1602 are found in diverse species in all kingdoms of life (Table 3). Strikingly, as in the case of *aatS*, genes encoding DUF1602 are often genetically associated with, and occasionally overlap, genes encoding transport systems. We conclude that *aatS* may encode a small protein that could be an ancillary subunit of the Aat transport system in ETEC.

**Table 3:** Phylogentic distribution of DUF1602-containing proteins.

Kingdom/Species	Number of DUF1602-containing proteins
***Archaea (all Euryarchaeota)***	**5**
***Bacteria***:*Actinobacteria*:*Chlamydiae*:*Cyanobacteria*:*Firmicutes*:*Proteobacteria*:	**146**202218100
**Eukaryotes***Fungi*:*Metazoa*:*Chlorophyta*:*Streptophyta*:	**59**141143
**Unclassified sequences**	**4**

## Discussion

In this study we have characterised the function of a predicted CRP binding site located between two convergent genes. We show that CRP activates transcription of *aatS*; a small ORF embedded within the much larger *aatC* gene. The *aatPABC* operon encodes a type I secretion system (the Aat system) found in many pathogenic bacteria [24, 25]. The presence of a functional RBS, correctly positioned upstream of *aatS*, suggests that the gene encodes a small protein rather than a regulatory RNA. Interestingly, many small proteins in bacteria localise to the membrane and function as accessory factors in transport systems [26]. Consistent with such a function, *aatS* is genetically associated with genes encoding transport systems in many bacteria. Furthermore, a potential transmembrane helix is predicted between residues 4 and 21 of AatS [27].

Documented instances of overlapping, protein-encoding, genes in bacteria are rare [28]. We are aware of only two examples; *rpmH* is encoded within *rnpA* in *Thermus thermophilus* and *setAB* resides inside the *pic* gene of *E*. *coli* 042 and *Shigella flexneri* [29, 30]. However, we speculate that further overlapping transcription units may become evident as unusual transcription factor targets are examined in detail. For instance, in a study of 154 *Mycobacterium tuberculosis* transcription factors, 75% of binding targets were located inside genes [31]. Similarly, a study of 116 transcription factors in *E*. *coli*, identified many intragenic binding events [32]. Presumably, some of these targets will control production of unannotated transcripts. Thus, in the case we have examined, the position of the CRP site is only surprising on first inspection. Detailed investigation of the *aat* locus reveals that CRP acts via a well-established mechanism and it is the position of *aatS*, embedded within *aatC*, which confounds the situation. In summary, whilst some bacterial transcription factors bind primarily in expected locations [33–35] many have unusually situated targets [3,10,11,36,37]. Surprising binding sites are often ignored [36] or dismissed as artefacts [38]. This work demonstrates that careful genetic and biochemical analysis can identify regulatory function for such targets.

## Supporting Information

S1 Fig Raw gel images. Panels A-C show raw gel images from Figs 2A, 2C and 3 respectively.
(PDF)
