# Correction: Unusually Situated Binding Sites for Bacterial Transcription Factors Can Have Hidden Functionality

**DOI:** 10.1371/journal.pone.0163074

**Published:** 2016-09-19

**Authors:** PLOS ONE staff

A formatting error was introduced into Table 3 during article production. Please view the correct table here. The publisher apologizes for the error.

**Table 3:** Phylogentic distribution of DUF1602-containing proteins

Kingdom/Species	Number of DUF1602-containing proteins
***Archaea (all Euryarchaeota)***	**5**
***Bacteria*:**	**146**
***Actinobacteria***	20
***Chlamydiae*:**	2
***Cyanobacteria*:**	2
***Firmicutes*:**	18
***Proteobacteria*:**	100
**Eukaryotes**	**59**
***Fungi*:**	14
***Metazoa*:**	1
***Chlorophyta*:**	1
***Streptophyta*:**	43
**Unclassified sequences**	**4**
