# Supplementary material for: Unusually Situated Binding Sites for Bacterial Transcription Factors Can Have Hidden Functionality
Source: PLoS One. 2016 Jun 3;11(6):e0157016. doi: 10.1371/journal.pone.0157016 (PMC4892627; doi:10.1371/journal.pone.0157016)
Supplement: S1 Fig — Panels A-C show raw gel images from Figs 2A, 2C and 3 respectively. (PDF) [file pone.0157016.s001.pdf]

Figure S1

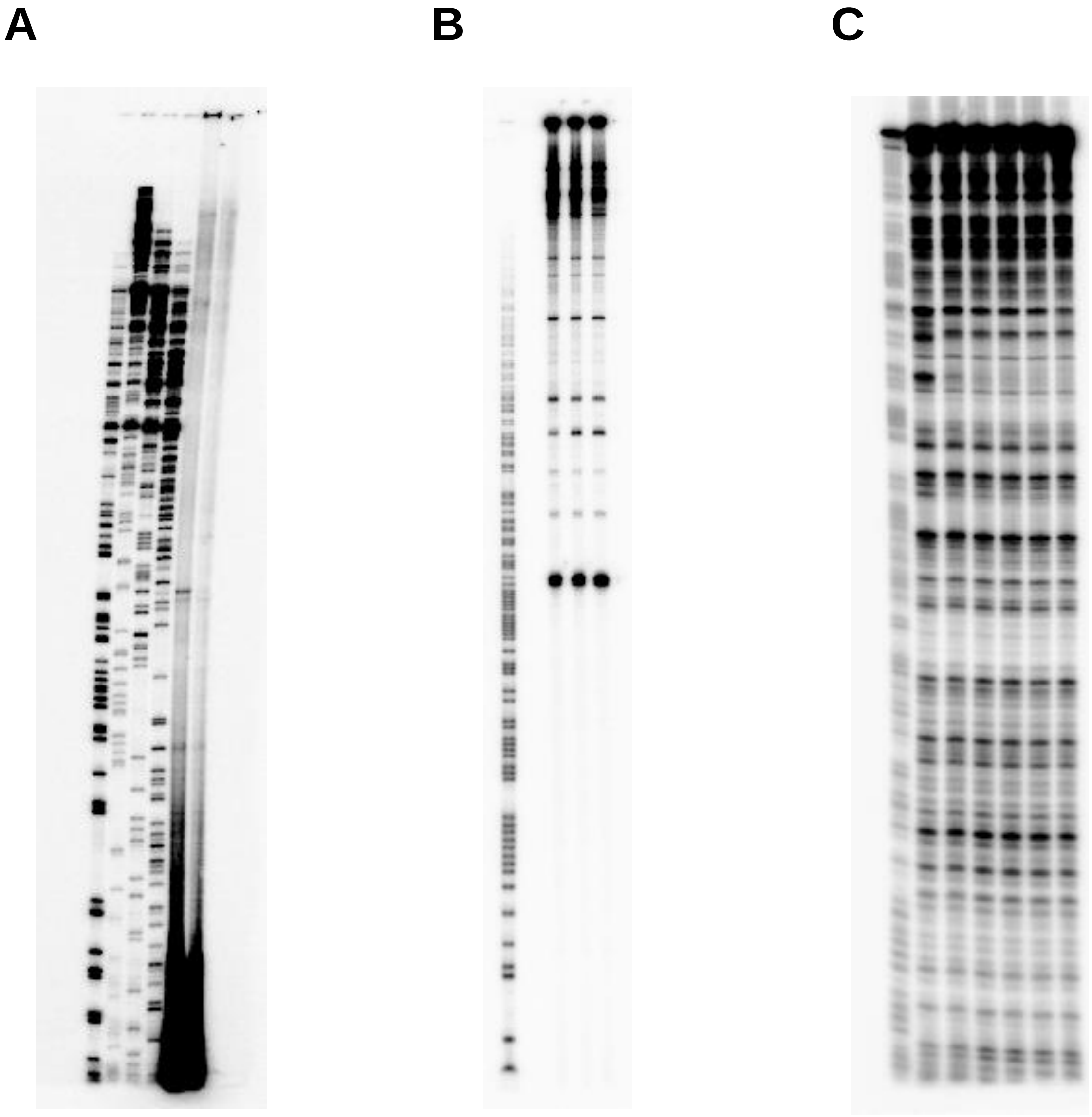

Figure S1: Raw gel images. Panels A-C show raw gel images from Figures 2A, 2C and 3 respectively.
